# Low-Coverage Whole Genome Sequencing Using Laser Capture Microscopy with Combined Digital Droplet PCR: An Effective Tool to Study Copy Number and Kras Mutations in Early Lung Adenocarcinoma Development

**DOI:** 10.3390/ijms222112034

**Published:** 2021-11-06

**Authors:** Elizabeth A. Mickler, Huaxin Zhou, Tzu L. Phang, Mark W. Geraci, Robert S. Stearman, Catherine R. Sears

**Affiliations:** 1 Division of Pulmonary, Critical Care, Sleep and Occupational Medicine, Department of Medicine, Indiana University School of Medicine, Indianapolis, IN 46202, USA; eamickle@iu.edu (E.A.M.); huaxzhou@iu.edu (H.Z.); rostearm@iu.edu (R.S.S.); 2 Division of Biomedical Informatics and Personalized Medicine, Department of Medicine, University of Colorado School of Medicine, Aurora, CO 80045, USA; tzu.phang@cuanschutz.edu; 3 Department of Medicine, University of Pittsburgh School of Medicine, Pittsburgh, PA 15261, USA; mgeraci@pitt.edu; 4 The Richard L. Roudebush Veterans Affairs Medical Center, Indianapolis, IN 46202, USA

**Keywords:** lung cancer, carcinogenesis, mouse, Kras, copy number alterations

## Abstract

Defining detailed genomic characterization of early tumor progression is critical to identifying key regulators and pathways in carcinogenesis as potentially druggable targets. In human lung cancer, work to characterize early cancer development has mainly focused on squamous cancer, as the earliest lesions are more proximal in the airways and often accessible by repeated bronchoscopy. Adenocarcinomas are typically located distally in the lung, limiting accessibility for biopsy of pre-malignant and early stages. Mouse lung cancer models recapitulate many human genomic features and provide a model for tumorigenesis with pre-malignant atypical adenomatous hyperplasia and in situ adenocarcinomas often developing contemporaneously within the same animal. Here, we combined tissue characterization and collection by laser capture microscopy (LCM) with digital droplet PCR (ddPCR) and low-coverage whole genome sequencing (LC-WGS). ddPCR can be used to identify specific missense mutations in Kras (Kirsten rat sarcoma viral oncogene homolog, here focused on Kras Q61) and estimate the percentage of mutation predominance. LC-WGS is a cost-effective method to infer localized copy number alterations (CNAs) across the genome using low-input DNA. Combining these methods, the histological stage of lung cancer can be correlated with appearance of Kras mutations and CNAs. The utility of this approach is adaptable to other mouse models of human cancer.

## 1. Introduction

Lung cancer remains a common and deadly disease, associated with a 5-year survival rate of only 19%, largely due to the majority of patients being diagnosed at a late stage when prolonged survival or cure are unlikely [1]. Lung cancer that is diagnosed at a pre-malignant or early malignant stage is associated with a high likelihood of cure, with the implementation of lung cancer screening using low-dose computed tomography associated with improved survival [2,3]. However, radiologic findings of pre-malignant and early lung cancers are often non-specific, with many inflammatory or infectious lung diseases presenting with similar radiologic characteristics. Pre-malignant growth patterns and metastatic potential are variable, with some pre-malignant lung lesions never progressing [4].

Lung cancer is broadly classified as either small cell lung cancer or the more common non-small cell lung cancer (NSCLC). Lung adenocarcinoma is the most common histologic type of NSCLC, with atypical adenomatous hyperplasia (AAH) being its only known precursor. AAH is believed to develop from SPC+ type 2 alveolar cells or, less commonly, from CC10+ Clara cells, and is characterized by an abnormal proliferation of type 2 alveolar-like cuboidal cells along the peripheral alveolar structure [5]. AAH is difficult to diagnose due to its small size (typically less than 5 mm diameter) and vague radiologic findings, which are often found incidentally in the lung tissue adjacent to surgically removed lung adenocarcinoma [6]. This has hampered extensive genotypic and molecular classifications of AAH and limited our understanding of the mechanisms underlying the evolution to AAH and to subsequent lung adenocarcinoma.

Kras is an important oncogene target in human lung cancer, as well as other cancers, including colorectal adenocarcinoma and pancreatic adenocarcinoma, with each having different Kras mutational profiles [7]. Several pre-clinical mouse and cell culture models have been developed to study lung adenocarcinoma development, each with their own strengths and limitations. Because Kras mutations are commonly found in lung adenocarcinoma, constitutive and conditional Kras mutant mouse models have been extensively studied. However, whether this represents early lung adenocarcinoma development is unclear, and recent studies have suggested that carcinogen-induced Kras mutations may differ between humans (typically G12C or G12D) and mouse models (Q61L and Q61R) [8,9]. Additional mouse models represent epidermal growth factor (EGFR) gene mutations known to be found in a subset of lung adenocarcinoma, but it is unclear if either of these models represent the early changes that are needed to progress to pre-malignant and ultimately malignant lung adenocarcinoma—particularly in tobacco smokers, who rarely harbor EGFR mutations [10]. Mouse models which utilize carcinogens, such urethane, that are found in cigarette smoke, represent potentially powerful tools to study lung adenocarcinoma development and progression. However, a greater understanding of these characteristic changes is needed.

Alterations in copy number alterations (CNA) and characteristic mutations in tumor suppressor and oncogenes are particularly prominent in lung adenocarcinomas but have also been described in adjacent non-cancerous bronchial epithelial specimens, highlighting the need to characterize critical genomic and transcriptomic changes associated with the earliest stages of carcinogenesis using physiologic relevant pre-clinical models [11,12,13]. Genomic instability due to altered DNA repair is a hallmark of cancer. Mice deficient in the DNA repair protein, Xeroderma Pigmentosum Group C (XPC), are particularly prone to urethane- induced lung adenocarcinoma and develop a range of histologically progressive lesions characteristic of human AAH to lung adenocarcinomas, often synchronously within the same mouse [14]. Others have studied genomic changes of lung adenocarcinomas in mice but, because of their small size, have relied on changes identified in the whole lung or in DNA pooled from multiple lung adenocarcinomas [8,15]. Here, we present methods for identification and characterization of Kras mutation status (digital droplet PCR, ddPCR) and CNAs (low-coverage whole genome sequencing, LC-WGS) respectively, on genomic DNA (gDNA) isolated by laser capture microscopy (LCM) from histologically-classified normal and lung adenocarcinoma lesions. These evaluations represent critical tools to study the development and progression of genomic changes in histologically characterized pre-malignant and early malignant lung adenocarcinomas.

## 2. Results

**Figure 1:**
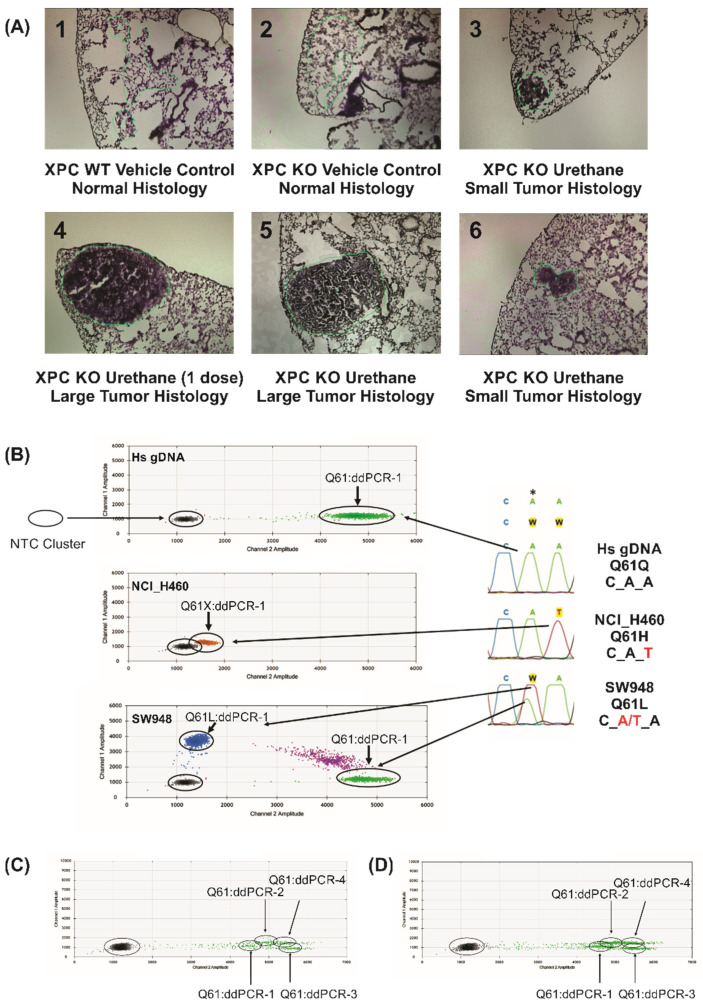
Differentiation of Kras Q61 mutants using digital droplet PCR. (**A**) Arcturus stained fresh-frozen lung slices of LCM samples used for this study, ranging from normal histology to large tumors, labelled 1A-1 to 1A-6 as indicated. 50× total magnification. (**B**) Sanger sequencing traces (right) of human gDNA from control and cancer cell lines (NCI-H460 and SW948) tested with the ddPCR-1 (Q61L) primer/probe assay. Homozygous WT Kras (Q61) was detected in control human gDNA (green, top). Expected mutations were found by ddPCR-1 assay in NCI-H460 (homozygous Q61H, CAt), which identified an aberrant ddPCR signal caused by a nearby unidentified mismatch Kras sequence (orange, middle). SW948 (heterozygous Q61L, CwA, w = A/T) showed both WT Kras (green, CAA) and mutant Kras Q61L (blue, CTA). Purple dots identify double-labelled droplets due to high gDNA input (not detected at significant levels using LCM gDNA inputs of 5–20 ng amplified gDNA). * Site of variant nucleotide in Kras PCR probes. (**C**) All four Kras Q61 ddPCR assays on LCM control normal sample 1A-1 (Xpc WT vehicle control), showing only WT Kras Q61 without mutations (CAA sequence). (**D**) All four Kras Q61 ddPCR assays on sample 1A-2 (LCM Xpc-/-, vehicle control), again showing only WT Kras Q61.

We completed an initial larger trial of LCM gDNA CNA (38 samples) using Affymetrix SNP-microarray, which has become limited by the availability of high-density SNP-microarrays and cost (data not shown). From this study, we selected six samples (Figure 1A) for testing Kras genotyping (ddPCR) and CNA (LC-WGS) from LCM gDNA.

Digital droplet PCR (ddPCR) was used to assess the mutation status and approximate percentage of cells harboring Kras Q61 codon changes. Instead of custom designing mouse primer/probe combinations, we explored the use of commercial Bio-Rad human Kras Q61 primer/probes designed for ddPCR. Looking at the homology of the human and mouse gDNA sequences in this region showed 100% identity 5′ and 3′ to Q61 for a length greater than expected to be needed in the ddPCR design. In addition, work by others has indicated aberrant ddPCR signals with sequence mismatches nearby the designed probe nucleotide position [16,17]. To ascertain these complexities, human lung cancer cell line control gDNAs were tested (Figure 1B). Each primer/probe assay is designed to detect a specific Kras Q61 mutation (Q61L, Q61R, Q61H 183A>T, Q61H 183A>C). In order to differentiate the assays more clearly from the mutations detected by each assay, we will subsequently refer to the primer/probe assays as ddPCR-1 through ddPCR-4, respectively, as detailed in Section 4. By way of example using ddPCR-1 (Bio-Rad Q61L primer/probe), human control gDNA found only signal in channel 2 (green, HEX) indicating only the wild-type (WT) Q61 CAA codon (Q61) was detected. Using ddPCR-1, the SW948 cell line gDNA (heterozygous Q61L, CaA->Ca/tT) produced strong WT (green, HEX), mutant (blue, FAM), and double-labelled droplets (purple) signals. Again, using ddPCR-1, NCI-H460 cell line gDNA (homozygous Kras Q61H, CAa->CAt) generated a strong aberrant signal (orange; Q61X), slightly offset from the no template control (NTC) signal with no detectable WT Q61 present. This was confirmed by ddPCR-3 to represent the expected Kras Q61H mutation (data not shown). Thus, the aberrant signal (orange; Q61X), slightly but reproducibly offset from the NTC signal, indicates a mutation nearby to the designed probe position. We then tested four different Q61 primer/probes (ddPCR-1 to ddPCR-4, as in Section 4) on the two normal histology LCM amplified mouse gDNA preparations (samples 1A-1 and 1A-2). In both cases, the normal histology LCM gDNA samples only contained the WT Kras Q61 sequence (Figure 1C,D).

**Figure 2:**
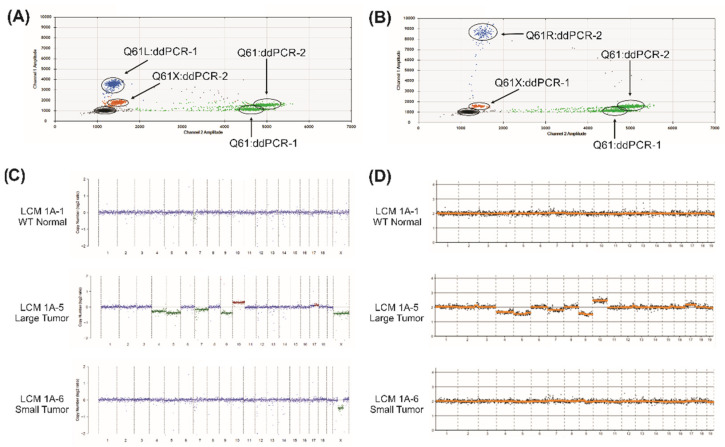
Typical ddPCR and CNA identified by LC-WGS. (**A**) Kras Q61 ddPCR-1 and ddPCR-2 assays on large tumor sample 1A-5 (Xpc-/-, urethane treated). ddPCR-1 assay (to detect Kras Q61L) shows both WT Kras (green) and Kras Q61L (blue) gDNA in this sample. ddPCR-2 assay (to detect Kras Q61R) confirms the nearby mismatch Kras sequence already identified as Kras Q61L by ddPCR-1 (orange signal). (**B**) Kras Q61 ddPCR-1 and ddPCR-2 assays on small tumor sample 1A-6 (Xpc-/-, urethane treated). ddPCR-2 assay (to detect Kras Q61R) shows a mixed gDNA composition of WT Kras (green) and Kras Q61R (blue) in this sample. ddPCR-1 assay (to detect Kras Q61L) confirms the nearby mismatch Kras sequence, already identified as Kras Q61R by ddPCR-2 (orange signal). (**C**) CNA analysis of LCM samples from LC-WGS data using ichorCNA to estimate copy number across the mouse genome including the sex chromosomes. Control LCM 1A-1 (top, no detected CNA), large tumor LCM sample 1A-5 (middle, extensive genome-wide CNA) and small tumor LCM sample 1A-6 (bottom, narrow deletion in X chromosome) are shown. (**D**) CNA analysis of LCM samples from LC-WGS data using ACE to estimate copy number across the mouse genome excluding the sex chromosomes. Control WT normal LCM 1A-1 (top) and the small tumor LCM sample 1A-6 (bottom) showed no detected CNA, while the large tumor LCM sample (1A-5) exhibited extensive genome-wide CNA (middle). Results from 1 Mb sequence bin size are shown.

The remaining four mouse LCM gDNA samples (summarized in Table 1) were tested. The results of ddPCR-1 and ddPCR-2 assays to detect Kras Q61L and Q61R mutations is shown (Figure 2A,B). The Q61L and Q61R are the most frequent Kras mutations in the mouse urethane model, while the third position wobble nucleotide Q61H is rarely found. Consistent with the published literature, we did not detect potential Kras Q61H mutations [8]. The results for Kras Q61L and Q61R in LCM gDNA sample 1A-5 indicate both WT Q61 (green) and mutant Q61L (blue), with the ddPCR-2 probe (designed to detect Q61R) producing the aberrant signal, as expected, for the Q61L mutant detected by ddPCR-2 (orange; Figure 2A). For sample 1A-6 (Figure 2B), using ddPCR-2 the mutation for Kras Q61R is detected (blue) supported by the aberrant signal (orange) from the ddPCR-1 probe (designed to detect Q61L). From the percentages of blue (mutant) and green (WT) signals, ddPCR can estimate the approximate measure of mutant copies present. This analysis does not distinguish at level of the individual cell’s two copy chromosomes whether it is heterozygous or homozygous. The two normal histology samples did not have detectable Kras Q61 mutations, while the other four tumor samples did, ranging from 14–51% Kras Q61 mutation content (Table 1).

The previous Affymetrix analysis indicated two samples with CNAs (Samples 1A-5 and 1A-6, Figure 1A). The other four samples, both tumor and normal histology (both WT and Xpc-/- mice), did not show CNA in this preliminary screening. LC-WGS was completed and the resulting .bam files were inputted into either ichorCNA [18] or ACE [19] (Figure 2C,D). Neither of the LCM normal gDNA samples (samples 1A-1 and 1A-2) showed CNAs with either analysis tool. The tumor samples (samples 1A-3 and 1A-4) also did not exhibit any CNAs (not shown) but, as summarized in Table 1, had Kras Q61 mutations detected by ddPCR (Q61L and Q61R, respectively). Tumor samples 1A-5 and 1A-6 had CNAs detected in addition to the Kras Q61 mutations (Figure 2C,D). Sample 1A-5 had a wide variety of large-scale CNA covering chromosomes 4, 5, 7, 8, and X (deletions), chromosome 10 (amplification), and was predicted to have ~40% tumor fraction. Sample 1A-6 showed a narrow deletion within chromosome X. The autosomal CNAs found in sample 1A-5 were reproduced using the ACE method, but the X chromosome CNAs are not analyzed on this platform. The webtool Ginkgo recapitulated these findings, including the X chromosome CNAs (data not shown).

## 3. Discussion

We demonstrated the potential of combining the tissue sample histology and LCM with ddPCR and LC-WGS to detect early genomic changes using the mouse urethane model of lung adenocarcinoma. In this study, we found differences in genomic composition within the four tumor samples tested. Both small and large tumors had CNAs along with either of the common Q61L or Q61R Kras mutations. Normal histology samples had no apparent CNAs or Kras mutations at Q61. Similar results were found using the ichorCNA and ACE methods, and either method is flexible in defining a read count bin size. The data presented here used a 1 Mb bin size. Decreasing the bin size has a trade-off of finer granularity for defining CNA genomic coordinates versus increased background noise. In addition, ichorCNA generates an output table based on its analysis (genomic coordinates, copy number changes, and estimated corrected p-value) and an estimated tumor fraction. We combined this with digital droplet PCR, which proved to be an effective method for determining Kras Q61 mutations and estimating the Kras mutation content of LCM samples. In our study, we used whole genome amplified LCM gDNA to both maximize the available input for LC-WGS and provide sufficient material for the multiple Kras mutations possible in lung cancer models. For the mouse Kras Q61 codon we were able to use the BioRad ddPCR products for human samples, due to the perfect homology between human and mouse genomes in this region. The region around Kras G12 is less conserved and in our initial testing, the BioRad human G12 products do not work well for mouse gDNA. However, others have published potential murine ddPCR primer/probes for this position [17]. Additional work to optimize the primer/probes for Kras G12 has high potential applicability, particularly in translational studies of Kras G12C-driven cancers, for which a first-in-class targeted therapy (sotorasib) has recently been approved for clinical use in non-small cell lung cancer [20]. Other sequencing approaches may be better suited for applications in which unbiased mutation discovery is desired, however, these typically require more starting material and may be cost-prohibitive. Our approach is particularly suited for identification and quantification of focused gene mutations combined with copy number alterations in samples with limited starting material, and provides a higher sensitivity than other established methodologies, such as fluorescence in situ hybridization (FISH). Finally, the ddPCR method is not limited to detecting point mutations in coding regions, but commercial mouse copy number primer/probes have been used to assess deletion/amplification of tumor suppressor or oncogene genes [21].

Tissue histology can be used to select samples within the same animal (germline genome identity) and across animals (different germline genomes) that cover the spectrum of observed changes during the development of lung adenocarcinoma. Though the Arcturus fresh frozen tissue staining does not give the detail found in traditional fixed H&E staining, we can differentiate normal tissue from early stage AAH, as well as small and larger tumors. Tumors can also be subdivided by apparent containment (adenoma) versus advanced in situ carcinoma. Future studies will expand the LCM samples to study the temporal development of CNA and Kras mutations from all stages of urethane-induced adenocarcinoma development to develop a detailed genomic model for mouse lung adenocarcinoma and in both Xpc deficient and proficient mice to study the impact of DNA repair deficiencies in this process. In addition, this approach can be expanded to other mouse carcinogen models, such as methyl-nitrosourea (MNU) which leads to a preferred G12D Kras mutation in lung adenocarcinoma, or mouse squamous lung cancer using N-nitroso-tris-(2-chloroethyl)urea (NTCU) where Tp53, Pten, Cdkn2, and Lkb1 deletion and/or mutation are potential key drivers [22]. Finally, these methods could be of particular value in targeted studies using human biopsy specimens, particularly those with limited tissue due to low accessibility or high risk of biopsy complications. In conclusion, combined LC-WGS with ddPCR provides a valuable tool to characterize genomic and mutational characteristics with histologic progression in pre-clinical models and in characterization of small and rare translational specimens.

## 4. Materials and Methods

### 4.1. Mouse and In Vivo Models of Lung Adenocarcinoma

Mouse breeding and urethane carcinogen models were performed as previously published, approved by Indiana University Institutional Biosafety Committee (IBC, IN-972) the Indiana University Animal Care and Use Committee (IACUC Protocol 18104) [14]. Briefly, female mice (Xpc WT and -/-, C57Bl/6;129 background aged six to eight weeks) were treated with urethane (1 g/kg body weight in PBS) or vehicle (PBS) weekly for six weeks. Necropsy was performed at 28 weeks after the initial urethane injection. Lung harvest and bronchoalveolar lavage (BAL) were performed as previously described with one lobe of the right lung frozen in TissueTek Optimal Cutting Temperature (O.C.T.) medium [14]. Human gDNA for ddPCR controls were obtained from Takara and ATCC cell lines (H460, SW948), and were confirmed as mycoplasma-free.

### 4.2. Laser Capture Microscopy (LCM) and Genomic DNA (gDNA) Purification and Amplification

Lung tissue frozen in O.C.T. was cut into seven serial sections, each 10µm thick. Using H&E staining on sections 1 and 7 as guides, the inter-leaved sections 2–6 were stained by Arcturus Histogene Frozen Section Staining Kit protocol (ABI KIT0419) and visually scored as either normal, hyperplasia, small tumor, or large tumor. At least four well-separated areas from each slide series were outlined and collected using LCM software (Leica LMD6 laser dissection system). From the fresh frozen LCM pieces, gDNA was prepared using the Arcturus PicoPure DNA extraction kit (ABI 11815-00) and quantitated by dsDNA Quantifluor fluorescent assay (Promega). Initial analyses involved amplification of 38 samples performed in duplicate for each LCM specimen using GenomiPhi whole genome amplification kits (Amersham), which was sent to Affymetrix’s contract services and run on a 96-well Axiom Mouse Diversity Genotyping Array plate. Subsequent copy number experiments did not require this initial amplification step and replicates were not performed.

### 4.3. Digital Droplet PCR (ddPCR)

GenomiPhi whole genome amplification kits (Amersham) were used for one round of amplification starting from 5–10 ng gDNA input yielding ~5 ug total LCM-amplified gDNA samples used for ddPCR. We used four human Kras mutation assays for Q61 from BioRad, subsequently referred to as ddPCR-1 to ddPCR-4. Those assays were: dHsaMDV2010101 Kras Q61L (ddPCR-1), dHsaMDV2010135 Kras Q61R (ddPCR-2), dHsaMDV2010133 Kras Q61H c.183A>C (ddPCR-3), dHsaMDV2010131 Kras Q61H c.183A>T (ddPCR-4). We followed the BioRad ddPCR workflow for probe chemistry. The LCM gDNA from the first amplification was diluted to 25 ng/µL in TE buffer and we used 50 ng in each assay. We used an annealing temperature of 55° C for all of the probes.

### 4.4. Low-Coverage Whole Genome DNA Sequencing (LC-WGS)

Five to 20 ng of unamplified LCM gDNA was submitted to the Indiana University Genomics Core for LC-WGS. TruPLEX tag-sequencing library kit (Takara) was used to both bar-code the individual samples, as well as incorporate Unique Molecular Identifiers (UMI). Because of the low input for WGS, UMIs are important to eliminate PCR-generated duplications. The libraries were run on an Illumina NextSeq (150 cycles, 2 × 75 bp Paired End (PE)) with the mixed libraries spread over two lanes. The samples were aligned to mm10 using bwa 0.7.12 [23]. Duplicate reads were identified and removed with connor v0.6.1 [24]. Prior to analysis, alignment statistics were assessed with samtools v1.5 and picard v2.18.2 [25]. Bed files were generated using bedtools v2.26.0. [26]. The targeted WGS sequencing coverage for this experiment was 0.5× coverage. For the six samples after de-duplication, the average PE reads remaining (5.9 M reads, 62% duplication) resulted in 0.2× WGS coverage.

Two different WGS copy number estimators were used to analyze the .bam files. The R package ichorCNA [25] was originally developed for CNA from LC-WGS from circulating cell free gDNA isolated from the blood of cancer patients. A second R package, ACE (Absolute Copy number Estimation [26]), was used as an alternative to ichorCNA, though as implemented, ACE does not consider the X-Y sex chromosomes due to their natural copy number differences. These two methods were chosen since they use different algorithms for copy number estimation. In addition, the webtool Ginkgo A web tool for analyzing single-cell sequencing data (Available online: http://qb.cshl.edu/ginkgo/, accessed on 6 November 2020) was tested on the derived .bed files for ease of use (simple web interface for data upload and analysis). All three tools provided comparable results. Data are available on request. Given the nature of our study, randomization, blinding, power analyses and statistical comparisons are not relevant.

## Figures and Tables

**Table 1:** Summary of Kras Q61 mutations and copy number variations found in mouse LCM samples modeling lung adenocarcinoma.

LCM Sample ID (Figure 1A Panel)	Sample Description	KRas Q61 Status	Percent Mutation	Copy Number Alterations
1A-1	WT vehicle control normal	Q61 (wt)	0%	None
1A-2	Xpc-/- vehicle control normal	Q61 (wt)	0%	None
1A-3	Xpc-/- urethane small tumor	Q61L	14%	None
1A-4	Xpc-/- urethane large tumor	Q61R	29%	None
1A-5	Xpc-/- urethane large tumor	Q61L	51%	Large Scale Alterations
1A-6	Xpc-/- urethane small tumor	Q61R	16%	X-Chromosome

## Data Availability

Data is available upon request to the authors.
